# The strategies and mechanisms of enteroviruses to evade innate immunity and the vaccine progress of enteroviruses

**DOI:** 10.3389/fcimb.2025.1636104

**Published:** 2025-07-31

**Authors:** Yangqi Yin, Xuyang Chi, Yandong Feng, Qinglian Jiang

**Affiliations:** ^1^ Department of Pediatrics, General Hospital of Fushun Mining Bureau of Liaoning Health Industry Group, Fushun, Liaoning, China; ^2^ Second Department of Internal Medicine, School of Medicine, University of Occupational and Environmental Health, Kitakyushu, Japan; ^3^ Department of Pediatrics, The First Hospital of Lanzhou University, Lanzhou, China; ^4^ Department of General Pediatrics, Zhongshan City People’s Hospital, Zhongshan, China

**Keywords:** enterovirus, innate immune response, immune evasion strategies, vaccine, interferon

## Abstract

Enteroviruses (EVs) are a group of highly contagious RNA viruses that can cause a wide range of diseases, from mild infections to severe complications like neurological disorders and myocarditis. This review focuses on the innate immune evasion strategies employed by EVs, highlighting their mechanisms and consequences. EVs evade host immune responses through various tactics, including inhibiting pathogen recognition receptors (PRRs) such as toll-like receptors (TLRs) and RIG-I-like receptors (RLRs), disrupting key signaling pathways like nuclear factor kappa-B (NF-κB) and (JAK)-signal transducers and activators of transcription (STAT), and directly targeting interferon (IFN) signaling components. Specific viral proteases, such as 2A protease (2A^pro^) and 3C protease (3C^pro^), play crucial roles in these evasion strategies by cleaving host proteins involved in immune signaling. Additionally, EVs manipulate host factors to suppress antiviral responses, exemplified by the upregulation of proteins like sex-determining region Y-box 4 (Sox4) and microRNAs (miRNAs) that inhibit TLR signaling. The review also discusses the development of vaccines against EVs, emphasizing the importance of prophylactic measures in controlling infections. Understanding these immune evasion mechanisms is essential for developing effective antiviral therapies and vaccines.

## 1 Introduction to EVs and innate immunity

Enteroviruses (EVs), belonging to the genus *Enterovirus*, are a group of RNA viruses classified within the *Picornaviridae* family. *Enterovirus* genus encompasses 15 distinct species. Among these, seven demonstrate human pathogenicity: four EV species (EV-A to EV-D) and three rhinovirus (RV) species (RV-A to RV-C) (Mbani et al., 2024). The EV group comprises highly contagious viruses that can lead to a wide spectrum of diseases, such as hand, foot, and mouth disease (HFMD), neurological disorders (e.g., encephalitis and aseptic meningitis), cardiac complications (e.g., myocarditis), ocular infections (e.g., acute hemorrhagic conjunctivitis), and respiratory and gastrointestinal infections. Although the majority of EV infections are subclinical or self-limiting, they may result in life-threatening complications in vulnerable populations including neonates, infants, and immunocompromised hosts (Khetsuriani et al., 2006; Xie et al., 2024).

EVs are non-enveloped viruses characterized by a positive-sense, single-stranded RNA genome enclosed within an icosahedral protein capsid (Rossmann, 1994). The EV capsid adopts an icosahedral symmetry, composed of 60 tightly packed protomers. Each protomer consists of four structural proteins: viral polypeptide 1 (VP1), VP2, VP3, and VP4. Among these, VP1-VP3 are surface-exposed, forming the outer capsid shell, while VP4 is internally positioned and functions as a structural stabilizer (Rossmann, 1994). The viral genome is a single-stranded RNA molecule ranging from 7,100 to 7,450 nucleotides (nt) in length (Kitamura et al., 1981). The genome features an open reading frame (ORF) bounded by structured 5’ and 3’ untranslated regions (UTRs), encoding a polyprotein processed into four structural (VP1-VP4) and seven nonstructural proteins (Wang et al., 2012). Recent genomic analyses have identified an additional open reading frame (ORF2) in certain EV strains, which encodes the ORF2p protein (Guo et al., 2019). This novel viral factor has been demonstrated to play a crucial role in facilitating viral replication within intestinal epithelial cells.

Through phylogenetic analysis of VP1 sequences, 116 distinct genotypes have been classified within the EV-A to EV-D groups. The distribution of these genotypes is as follows: EV-A contains 25 genotypes, EV-B encompasses 63 genotypes, EV-C includes 23 genotypes, and EV-D comprises 5 genotypes (Nix et al., 2006; Liu, 2017; Simmonds et al., 2020).

EVs infection produces pathogen-associated molecular patterns (PAMPs) that are detected by epithelial pattern recognition receptors (PRRs), including toll-like receptors (TLRs), retinoic acid-inducible gene I (RIG-I)-like receptors (RLRs), and nucleotide-binding oligomerization domain (NOD)-like receptors (NLRs) (Wei et al., 2024). Upon viral RNA recognition, TLRs activate immune responses through two distinct signaling cascades: the myeloid differentiation primary response protein 88 (MyD88)-mediated pathway and the toll/interleukin (IL)-1 receptor domain-containing adaptors inducing interferon (IFN)-β (TRIF)-dependent pathway (Takeda and Akira, 2015). TLR7/9 engagement initiates MyD88-dependent signaling through death domain-mediated recruitment of IL-1 receptor-associated kinase 4 (IRAK4), which phosphorylates IRAK1. Activated IRAK-1 subsequently binds tumor necrosis factor receptor-associated factor 6 (TRAF6), triggering downstream cascades that ultimately induce nuclear factor kappa-B (NF-κB) nuclear translocation and inflammatory gene expression (Takeda and Akira, 2004). Conversely, TLR3 activates a distinct TRIF-dependent pathway where TRIF recruits TRAF3 to scaffold TANK-binding kinase 1(TBK1)/inhibitor of κB kinase ϵ (IKKϵ) complexes. These non-canonical IκB kinases phosphorylate interferon (IFN) regulatory factors (IRF) 3/7, driving type I IFN production (Takeda and Akira, 2015; Ma et al., 2023). RLRs recruit mitochondrial antiviral-signaling protein (MAVS) (Hou et al., 2011), which then engages TRAF3 and TRAF6 via its proline-rich region domain. This interaction triggers the activation of both the TBK1 and IKK complexes, initiating downstream antiviral signaling (Ren et al., 2020).

The third major PRR family comprises NLRs, known to play a central role in mediating inflammatory responses against viral infections. NLRs primarily function as inflammasome sensors that detect both PAMPs and damage-associated molecular patterns (DAMPs) (Hu and Chai, 2023). This recognition triggers inflammasome assembly, leading to caspase-1 activation and subsequent maturation of IL-1β, IL-18, and gasdermin D (GSDMD), thereby driving inflammatory responses and pyroptotic cell death (Chou et al., 2023). The 2B protease (2B^pro^) encoded by multiple EVs species directly interacts with NLR pyrin domain containing 3 (NLRP3), facilitating the recruitment and subsequent oligomerization of apoptosis-associated speck-like protein containing a caspase activation and recruitment domain (CARD) adaptor protein (ASC). This interaction promotes NLRP3 inflammasome assembly and activation, ultimately inducing IL-1β maturation and secretion while initiating pyroptotic cell death (Wang et al., 2022). The 3C^pro^ of multiple EVs, including RV, coxsackievirus B3 (CV-B3), and EV-A71, specifically cleave human NLRP1, thereby activating the NLRP1 inflammasome and subsequently promoting the secretion of proinflammatory cytokines including IL-1β and IL-18 (Robinson et al., 2020; Tsu et al., 2021).

Cyclic GMP-AMP synthase (cGAS), a newly characterized PRR, detects cytoplasmic viral DNA and mitochondrial DNA, serving as a crucial mediator of innate antiviral immune responses (Sun et al., 2013). Notably, certain EVs, including EV-A71, EV-D68, and CV-A16, induce mitochondrial damage during infection. The resulting release of mitochondrial DNA (mtDNA) activates the cGAS-stimulator of IFN genes (STING) pathway, triggering IFN production (Zheng et al., 2023). Furthermore, TRAF3 has been identified as a critical mediator in this antiviral signaling cascade. A separate study indicates that the 2B^pro^ of EV-A71 and CV-A16 triggers mitochondrial permeability transition pore (mPTP) opening, leading to mtDNA release, which activates the cGAS-STING pathway and subsequently enhances type I IFN production, thereby exerting antiviral effects (Liu et al., 2023).

Functioning as key mediators of antiviral immunity, IFNs exert their protective effects via specific receptor complexes: type I (type I IFN receptor 1 (IFNAR1) and type I IFN receptor 2 (IFNAR2)), type II (IFN-γ receptor 1(IFNGR1) and IFN-γ receptor 2 (IFNGR2)), and type III (IFN-λ receptor 1 (IFNLR1) and IL-10 receptor 2 (IL-10R2)) (Schroder et al., 2004; De Weerd et al., 2007; Zhou et al., 2011). These receptors initiate Janus activated kinase (JAK)-signal transducers and activators of transcription (STAT) signaling cascades that ultimately induce the expression of hundreds of interferon-stimulated genes (ISGs), thereby establishing a multifaceted antiviral state (Wei et al., 2024). For example, the type I IFN induces protein kinase R (PKR) and oligoadenylate synthetase (OAS) expression, which collectively mediate antiviral defense through distinct mechanisms: PKR-mediated eukaryotic translation initiation factor 2 (eIF-2α) phosphorylation halts viral translation (Gao et al., 2022), while OAS-dependent ribonuclease L (RNase L) activation cleaves cytosolic viral RNA (Drappier and Michiels, 2015). The resulting viral RNA cleavage products activate melanoma differentiation-associated antigen 5 (MDA5) (a member of the RLR family), triggering IFN production (Chakrabarti et al., 2011).

## 2 Evasion of PRRs detection

### 2.1 Evasion of TLRs

**Figure 1:**
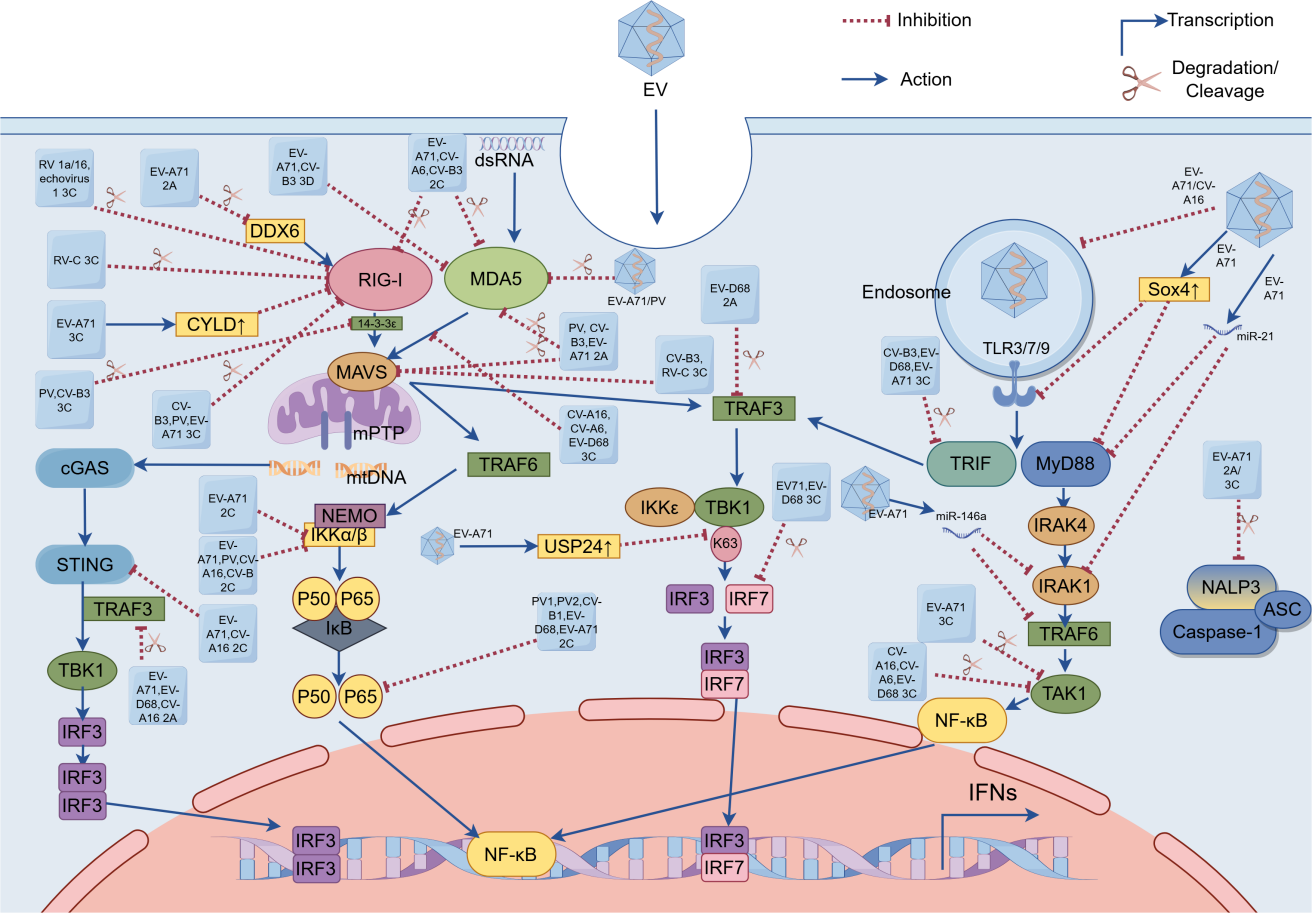
Enterovirus (EV) evasion strategies of pattern recognition receptor (PRR)-mediated signaling pathways. These EVs (e.g. EV-A71, EV-D68, poliovirus, coxsackievirus, rhinovirus, and echovirus) evade innate immunity by utilizing viral proteases (e.g. (1) 2A^pro^ encoded by EV-A71, EV-D68, poliovirus, and coxsackievirus; (2) 2C^pro^ encoded by EV-A71, EV-D68, poliovirus, and coxsackievirus; (3) 3C^pro^ encoded by EV-A71, EV-D68, poliovirus, coxsackievirus, rhinovirus, and echovirus; and (4) 3D^pol^ encoded by EV-A71 and coxsackievirus), host cellular factors (e.g. USP24, Sox4 and CYLD), and microRNAs to target PRRs (including TLRs, RLRs, and NLRs), adaptor proteins (including MAVS, TRIF and MyD88), and key downstream signaling effectors(e.g. TRAF3, TRAF6, IRF7 and NF-κB) and kinases (e.g. IRAF4, IKKs, TBK1 and TAK1). EVs inhibit cGAS-STING signaling via: 2C^pro^-mediated STING-TBK1 disruption (EV-A71/CV-A16) and 2A^pro^-dependent TRAF3 cleavage (EV-A71/EV-D68/CV-A16), collectively suppressing IRF3 activation. EV, Enterovirus; dsRNA, double-stranded RNA; EV-A71, Enterovirus-A71; EV-D68, Enterovirus-D68; PV, poliovirus; CV-A6, coxsackievirus-A6; CV-A16, coxsackievirus-A16; CV-B, coxsackievirus-B; CV-B3, coxsackievirus-B3; RV 1a/16, rhinovirus 1a/16; RV-C, rhinovirus-C; 2A, 2A protease; 2C, 2C protease; 3C, 3C protease; 3D, 3D polymerase; TLR3/7/9, toll-like receptor; RIG-I, RIG-I-like receptor; MDA5, melanoma differentiation-associated antigen 5; TRIF, Toll/interleukin (IL)-1 receptor domain-containing adaptor-protein-inducing interferon-β; MyD88, myeloid differentiation primary-response protein 88; MAVS, mitochondrial antiviral signaling protein; IRAK1/4, IL-1 receptor-associated kinase 1/4; mitochondrial DNA, mtDNA; cyclic GMP-AMP synthase, cGAS; STING, stimulator of interferon genes; NLRP3, NOD-like receptor (NLR) family pyrin domain-containing 3; apoptosis-associated speck-like protein containing a caspase activation and recruitment domain, ASC; DDX6, DEAD-box helicase 6; CYLD, cylindromatosis (CYLD); USP24, ubiquitin-specific protease 24; Sox4, sex-determining region Y-box 4; TBK1, TANK-binding kinase 1; TAK1, transforming growth factor β-activated kinase 1; IκB, Inhibitors of NF-κB; IKKα/β, inhibitor of kappa B kinase α/β; IKKϵ, inhibitor of kappa B kinase ϵ; NF-κB, nuclear factor kappa-B; IRF 3/7, interferon regulatory factor 3/7; IFNs, interferons.

EVs have evolved sophisticated strategies to subvert TLR-mediated antiviral immunity through multiple mechanisms. EV-A71 orchestrates transcriptional suppression by upregulating sex-determining region Y-box 4 (Sox4), which binds promoters of most *TLR* genes (excluding *TLR2*) and *MyD88*, broadly inhibiting TLR responses (Shang et al., 2021). In human bronchial epithelial (16HBE) cells, EV-A71 and CV-A16 infection induces autophagy-mediated disruption of endosomal trafficking, resulting in decreased TLR7 expression and compromised type I IFN production (Song et al., 2018). EVs systematically disable TLR-mediated antiviral responses through targeted disruption of downstream signaling effectors. The 3C^pro^ of CV-B3, EV-D68, and EV-A71 mediate proteolytic cleavage of TRIF, a critical adaptor molecule in TLR3 signaling, thereby attenuating downstream signal transduction (Lei et al., 2011; Mukherjee et al., 2011; Xiang et al., 2014). In parallel, EV-A71 manipulates host microRNAs (miRNAs) through multiple mechanisms: infection induces miR-21 upregulation, which directly targets both MyD88 and IRAK1 to suppress TLR signaling (Feng et al., 2017); promotes selective packaging of miR-30a into exosomes from infected oral epithelial cells, which subsequently deliver this inhibitory miRNA to macrophages to attenuate type I IFN responses through MyD88 suppression (Wang et al., 2020); and elevates miR-146a expression, thereby attenuating host antiviral responses via miR-146a-mediated suppression of critical TLR adaptors IRAK1 and TRAF6 (Ho et al., 2014).EV-D68 2A^pro^ disrupts TLR3-mediated IFN-β induction by cleaving TRAF3, thereby preventing TBK1/IKKϵ recruitment and subsequent IRF3/IRF7 phosphorylation in the TRIF-dependent pathway (Kang et al., 2021). Notably, transforming growth factor β-activated kinase 1 (TAK1) serves as a critical signaling hub linking pathogen recognition to NF-κB activation, primarily through IKK complex phosphorylation. However, EVs subvert this node via 3C^pro^-mediated cleavage: CV-A16, CV-A6, and EV-D68 3C^pro^ directly degrade TAK1 (Rui et al., 2017). This evasion strategy, which is also employed by EV-A71 3C^pro^, was further elucidated in a recent study showing that the protease cleaves the TAK1 complex to inhibit NF-κB activation (Lei et al., 2014). Beyond proteolytic cleavage, Sox4 suppresses innate immunity by dually inhibiting kinase activation—not only attenuating IRAK4/TAK1 in MyD88-dependent TLR signaling but also impairing TBK1 phosphorylation in TRIF-dependent cascades, thereby broadly blocking NF-κB and IRF3 (Shang et al., 2021). Additionally, EV-A71 exploits post-translational modification, infection-induced ubiquitin-specific protease 24 (USP24) reduces K63-linked ubiquitination of TBK1, crippling its ability to activate IRF3 (Zang et al., 2023). Collectively, these complementary evasion tactics illustrate how EVs employ a multi-pronged approach to paralyze TLR-dependent immune surveillance pathways (**Figure 1**).

### 2.2 Evasion of RLRs

The RLRs, including RIG-I and MDA5, are cytoplasmic RNA sensors that play a critical role in detecting enterovirus infections. While MDA5 exhibits preferential binding to long double-stranded RNA (dsRNA) (Onomoto et al., 2021), RIG-I demonstrates selective recognition of shorter dsRNA molecules (≥10 bp) featuring 5’-triphosphate (5’-ppp) or 5’-diphosphate (5’-pp) groups (Kell and Gale, 2015). However, EVs evade RIG-I detection by covalently attaching the viral protein genome-linked (VPg) peptide to the 5’ end of their RNA, thereby masking the 5’-ppp required for RIG-I recognition (Yoneyama and Fujita, 2009). Therefore, it is often believed that the vast majority of EV infections activate MDA5 rather than RIG-I (Feng et al., 2012). However, emerging evidence indicates that RIG-I-mediated recognition is indispensable for type I IFN induction following CV-B3 infection (Francisco et al., 2019).

EVs have evolved sophisticated mechanisms to subvert host antiviral defenses by specifically targeting the RNA sensors MDA5 and RIG-I. First, regarding MDA5 disruption, distinct EV species employ different proteolytic strategies: while Poliovirus (PV) uniquely induces MDA5 degradation through both proteasomal and caspase-dependent pathways (Barral et al., 2007). EV-A71 likely triggers MDA5 cleavage via caspase activation (Kuo et al., 2013). Furthermore, viral 2C protease (2C^pro^) from EV-A71, CV-A6, and CV-B3 specifically directs MDA5 to lysosomal degradation (Wang et al., 2023), whereas the 3C^pro^ encoded by CV-A16, CV-A6, and EV-D68 binds MDA5 to prevent MAVS association without affecting protein abundance (Rui et al., 2017). Additionally, CV-B3, EV-A71, and PV utilize their 2A^pro^ to cleave and inactivate MDA5 (Feng et al., 2014), and interestingly, EV-A71-encoded the RNA-dependent RNA polymerase (RdRP; also called 3D^pol^) targets the CARD of MDA5 to inhibit IFN-β production, a strategy shared by CV-B3 which employs its 3D^pol^ to similarly impair MDA5-mediated antiviral responses (Kuo et al., 2019). Transitioning to RIG-I targeting mechanisms, EVs employ both direct and indirect approaches. Direct proteolytic cleavage by viral 3C^pro^ represents a common mechanism shared by CV-B3, PV, and EV-A71, which physically cleaves RIG-I to prevent viral RNA detection (Feng et al., 2014). Another study revealed that the 3C^pro^ of RV 1a/16 and echovirus 1 similarly cleave RIG-I, though the precise cleavage sites remain unidentified (Barral et al., 2009). Meanwhile, the viral 2C^pro^ of EV-A71, CV-A6, and CV-B3 facilitate RIG-I degradation through the host lysosomal pathway (Wang et al., 2023). RV-C 3C^pro^ induces caspase-dependent degradation of RIG-I, effectively suppressing this critical viral RNA sensor (Pang et al., 2017). Additionally, EV-A71 has evolved a more sophisticated indirect strategy involving host factor manipulation, it upregulates cellular deubiquitinase cylindromatosis (CYLD) expression to catalytically remove the essential K63-linked ubiquitin chains from RIG-I, thereby suppressing its ability to activate type I IFN production (Xu et al., 2014). EV-A71-encoded 2A^pro^ mediates proteolytic cleavage of host DEAD-box helicase 6 (DDX6), effectively suppressing DDX6’s positive regulatory role in RIG-I-dependent type I IFN production (Zhang et al., 2021). During RIG-I activation, 14-3-3ϵ serves as a molecular escort that guides RIG-I to mitochondria, where MAVS interaction occurs and downstream signaling cascades are initiated (Liu et al., 2012). The 3C^pro^ of PV and CV-B3 mediate proteolytic cleavage of 14-3-3ϵ, thereby disrupting its chaperone function and impairing RIG-I’s ability to recruit downstream adaptor proteins (Andrews et al., 2023). Moreover, recent research indicates that CV-B3 upregulates the host miR-30a to enhance its own replication. MiR-30a targets tripartite motif protein 25 (TRIM25), effectively suppressing type I IFN signaling. This inhibition of TRIM25 and its mediation of RIG-I ubiquitination ultimately leads to reduced IFN-β activation and production, thereby promoting CV-B3 replication (Li et al., 2020). The 3D^pol^ of EV-D68 mediates the downregulation of phosphoglycerate mutase 5 (PGAM5), leading to a consequent upregulation of mitofusin 2 (MFN2) protein levels. This mitochondrial reprogramming exerts dual inhibitory effects on host defense mechanisms: it disrupts normal mitochondrial dynamics and function, while simultaneously impairing RIG-I receptor signaling pathway activation (Yang et al., 2021). Finally, beyond targeting RLR itself, EVs disrupt innate immune signaling by cleaving or degrading key adaptor molecules (such as MAVS), effectively blocking signal transduction and promoting immune escape. Notably, the 2A^pro^ of PV, CV-B3, and EV-A71 mediate proteolytic cleavage of MAVS (Feng et al., 2014). Additionally, CV-B3 and RV-C 3C^pro^ also targets MAVS for degradation (Mukherjee et al., 2011; Pang et al., 2017) (**Figure 1**).

### 2.3 Evasion of NLRs

EVs employ multiple molecular strategies to evade innate immune surveillance by specifically targeting NLRs. The viral 2A^pro^ and 3C^pro^ of EV-A71 specifically cleave NLRP3 at distinct sites (2A^pro^: G493-L494; 3C^pro^: Q225-G226), while the 3C^pro^ additionally interacts with NLRP3 to potently inhibit IL-1β secretion (Wang et al., 2015). EV-A71 has evolved additional immune evasion mechanisms by specifically targeting downstream effectors of the NLR signaling pathway. Pyroptosis serves as an effective antiviral mechanism that suppresses EV-A71 replication, GSDMD_1–275_ being the critical executor of this programmed cell death pathway. However, EV-A71 has evolved an immune evasion strategy through its 3C^pro^-mediated cleavage of GSDMD. The resulting GSDMD_1–197_ loses its pyroptosis-inducing capacity, thereby enabling viral immune escape (Lei et al., 2017).

### 2.4 Suppression of cGAS-STING pathway

The 2C^pro^ of EV-A71 and CV-A16 directly binds to STING, disrupting its interaction with TBK1 and consequently suppressing activation of the cGAS-STING signaling pathway (Liu et al., 2023). The 2A^pro^ of EV-A71 suppresses STING-TBK1 signaling by cleaving TRAF3, inhibiting TBK1 and IRF3 phosphorylation (Zheng et al., 2023). This STING-inhibitory function is shared by the 2A^pro^ of EV-D68 and CV-A16 (**Figure 1**).

## 3 Disruption of downstream effectors in innate immune signaling pathways

NF-κB serves as a master regulator of virus-induced inflammation. The IKK complex—comprising catalytic subunits IKKα/IKKβ and the regulatory component NF-κB essential modulator (NEMO, also known as IKKγ)—precisely controls NF-κB activation through phosphorylation-dependent degradation of Inhibitors of NF-κB (IκB) (Barnabei et al., 2021). The 2C^pro^ of EV-A71, PV, CV-A16, and CV-B recruit protein phosphatase 1 (PP1) to form a ternary 2C-PP1-IKKβ inhibitory complex that suppresses NF-κB signaling through inhibiting IKKβ phosphorylation (Li et al., 2016). EV-A71 2C^pro^ exploits IKKβ as a scaffold to compartmentalize IKKα into viral inclusion bodies (IBs), thereby disrupting NF-κB signaling without direct IKKα interaction (Ji et al., 2021). The p65/p50 heterodimer represents the predominant and functionally critical NF-κB configuration. Viral 2C^pro^ from PV1, PV2, CV-B1, EV-D68, and EV-A71 allosterically disrupt heterodimer formation through specific interactions with the IPT domain of p65 (Du et al., 2015). EV-A71-mediated Sox4 expression inhibits IKKα/β kinase activity via TAD domain binding, resulting in decreased IκBα phosphorylation and delayed NF-κB nuclear translocation (Shang et al., 2021). Both EV-A71 and EV-D68 employ their 3C^pro^ to cleave IRF7, thereby suppressing interferon production (Lei et al., 2013; Xiang et al., 2016). However, their 3C^pro^ recognizes different cleavage sites on IRF7 (**Figure 1**).

## 4 Antagonizing both IFN response and ISG products

**Figure 2:**
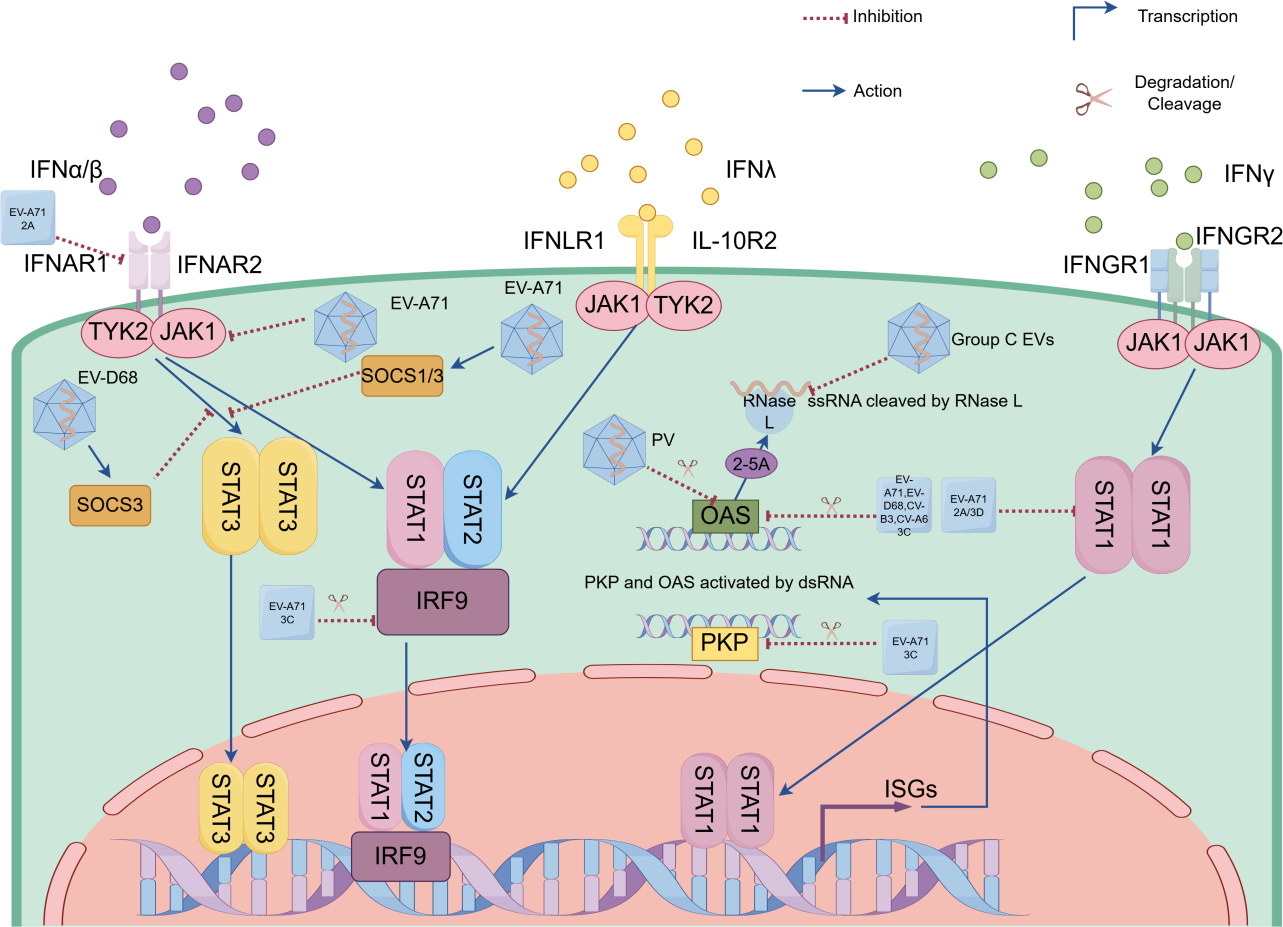
Enteroviruses (EVs) evade innate immunity by antagonizing interferon responses and suppressing interferon-stimulated gene (ISG) product functions. EVs (e.g. EV-A71, coxsackievirus and EV-D68) evade interferon (IFN) responses through viral proteases (e.g. 2A^pro^ encoded by EV-A71, 3C^pro^ encoded by EV-A71, coxsackievirus and EV-D68, as well as 3D^pol^ encoded by EV-A71) and host SOCS proteins that suppress IFN receptor and key components of Janus activated kinase (JAK)-signal transducers and activators of transcription (STAT) signaling pathways. EVs suppress ISG product functions via distinct strategies: (1) 3C^pro^-mediated cleavage of OAS in EV-A71, EV-D68, CV-B3 and CV-A6 infections; (2) PKR degradation through either EV-A71 3C^pro^ or poliovirus; and (3) Group C EVs RNA structural motifs that competitively inhibit RNase L enzymatic function. EV-A71, Enterovirus-A71; EV-D68, Enterovirus-D68; PV, poliovirus; CV-A6, coxsackievirus-A6; CV-B3, coxsackievirus-B3; Group C EVs, Group C Enteroviruses; 2A, 2A protease; 3C, 3C protease; 3D, 3D polymerase; IFN-α/β, interferon α/β; IFN-λ, interferon λ; IFN-λ, interferon λ; IFNAR1/2, type I interferon receptor 1/2; IFNAR1/2, type I interferon receptor 1/2; IFNGR1/2, IFN-γ receptor 1/2; IFNLR1, IFN-λ receptor 1; IL-10R2, interleukin (IL)-10 receptor 2; JAK1, Janus kinase 1; TYK2, tyrosine kinase 2; SOCS1/3, suppressor of cytokine signaling 1/3; STAT1/2/3, signal transducer and activator of transcription 1/2/3; IRF 9, interferon (IFN) regulatory factor 9; ZAP, zinc-finger antiviral protein; PKR, protein kinase R; OAS, oligoadenylate synthetase; RNase L, ribonuclease L; ssRNA, single-stranded RNA; dsRNA, double-stranded RNA; 2-5A, 2’-5’ oligoadenylate; ISGs, interferon-stimulated genes.

EVs employ diverse strategies to evade host antiviral responses, particularly through interference with IFN-mediated signaling. EV-A71 demonstrates multiple approaches to inhibit type I IFN-mediated signaling. One key mechanism involves the viral 2A^pro^, which reduces IFNAR1 levels in a protease-dependent manner by upregulating LDL-receptor-related protein-associated protein 1 (LRPAP1), a ligand that binds IFNAR1’s extracellular domain, promoting its degradation and ubiquitination (Lu et al., 2012; Li et al., 2023). However, studies in human embryonic lung fibroblasts and rhabdomyosarcoma cells reveal an alternative pathway: EV-A71 infection suppresses IFN-mediated signaling by downregulating JAK1 independently of viral 2A^pro^ and 3C^pro^ or the cellular proteasome (Liu et al., 2014). The formation of the ISG Factor 3 (ISGF3) complex (comprising phosphorylated STAT1, STAT2, and IRF9) is critical for IFN signaling, but EV-A71 3C^pro^ cleaves IRF9, disrupting this complex (Hung et al., 2011; Nowicka et al., 2023). Additionally, STAT1 nuclear translocation relies on karyopherin-α1 (KPNA1), which EV-A71 degrades via caspase3 activation (Wang et al., 2017). While both 2A^pro^ and 3D^pol^ impair IFNγ signaling by blocking STAT1 nuclear transport, their mechanisms differ: 2A^pro^ reduces STAT1 expression, whereas 3D^pol^ diminishes its phosphorylation (Wang et al., 2015). Notably, other EVs like PV and EV-D68 share similar immune evasion strategies, employing 3C^pro^ to cleave STAT1 and block its nuclear translocation, thereby inhibiting JAK/STAT signaling (Li et al., 2024). Further modulating immune responses, EV-A71 exploits the suppressor of cytokine signaling (SOCS) proteins—endogenous inhibitors of JAK/STAT signaling. Early infection triggers SOCS1/3 expression via the NF-κB pathway, suppressing STAT3 phosphorylation and thereby dampening IFN-mediated antiviral defenses (Linossi and Nicholson, 2015; Gao et al., 2020). In contrast, EV-D68 has evolved an alternative mechanism involving upregulation of the transcriptional regulator regulatory factor X 7 (RFX7), which specifically enhances SOCS3 expression to inhibit STAT3 phosphorylation and subsequent IFN-β-induced ISG products (Zhang et al., 2023) (**Figure 2**).

EVs have evolved sophisticated strategies to counteract the antiviral functions of ISG products, thereby enhancing their replication efficiency. One such ISG transcription protein, the zinc-finger antiviral protein (ZAP), demonstrates robust antiviral activity against EV-A71. However, EV-A71 effectively evades this defense mechanism by utilizing its 3C^pro^ to cleave ZAP in a protease-dependent manner. This cleavage generates non-functional fragments that no longer inhibit viral replication (Xie et al., 2018). Moreover, the 3C^pro^ of several EVs, including EV-A71, EV-D68, CV-B3, and CV-A6, but not CV-A16, mediates the proteolytic cleavage of OAS3. Specifically, EV-A71’s 3C^pro^ targets OAS3 at the Gln982-Gly983 site, thereby disrupting its antiviral function (Zhou et al., 2022). Similarly, EV-A71’s 3C^pro^ cleaves PKR at Gln188-Ser189, producing an N-terminal fragment that, counterintuitively, promotes viral replication (Chang et al., 2017). While PV also degrades PKR, the precise mechanism underlying this process remains to be elucidated (Black et al., 1989; Black et al., 1993). RNase L, an interferon-inducible antiviral effector, exists as an inactive monomer until viral infection triggers OAS-mediated 2’-5’ oligoadenylate (2-5A) production, which binds its ankyrin repeats to induce active dimerization and subsequent viral RNA degradation (Drappier and Michiels, 2015). Group C EVs utilize phylogenetically conserved RNA structural motifs that specifically impair RNase L’s catalytic function through competitive inhibition, while maintaining the enzyme’s ability to bind 2-5A (Townsend et al., 2008) (**Figure 2**).

## 5 The vaccine progress of EVs

In the context of limited therapeutic options against the full spectrum of EV-induced diseases—from mild presentations to lethal cases—prophylactic vaccination emerges as the primary intervention for infection control. Polio vaccination efforts have achieved remarkable success in reducing the global disease burden. In 1988, approximately 350,000 cases were reported across 125 endemic countries. By 2012, the Americas, Western Pacific, and European regions had been certified as polio-free, with worldwide cases declining to just 650— representing a reduction exceeding 99% (M A, 2022). Currently, all remaining wild poliovirus cases globally are caused by serotype 1, while wild poliovirus types 2 and 3 have been officially declared eradicated (M A, 2022; Bandyopadhyay et al., 2024). Wild PV type 1 (WPV1) transmission persists in only a few endemic countries, predominantly Afghanistan and Pakistan. Surveillance data show these two nations reported 22 WPV1 polio cases in 2022, which declined to 12 cases in 2023 (Geiger et al., 2024). Polio-free nations must remain vigilant against potential resurgence. Systematic wastewater monitoring in five European nations (Finland, Germany, Poland, Spain, and UK) has identified poliovirus circulation since September 2024. While no paralytic cases have been reported, these environmental findings demonstrate the continued risk of poliovirus transmission worldwide (Esposito and Principi, 2018). The two polio vaccine formulations—oral polio vaccine (OPV, live-attenuated) and inactivated polio vaccine (IPV)—differ fundamentally in their protective mechanisms. OPV has a unique ability to replicate in the intestinal tract and induce superior mucosal immunity, making it significantly more effective than IPV at preventing wild-type virus transmission (Burns et al., 2014). However, OPV carries the risk of generating circulating vaccine-derived PVs (cVDPVs) through mutation and reversion to neurovirulent strains during intestinal replication (Burns et al., 2014). To address this issue, a novel oral type 2 polio vaccine (nOPV2) strain has been developed. This strain features targeted modifications to the Sabin genome, including structural optimization of the 5’-untranslated region (UTR) and fidelity-enhancing mutations in the viral 3D^pol^ (Yeh et al., 2020). These modifications collectively restrict viral evolutionary capacity while preventing reversion to neurovirulence (Yeh et al., 2020). HFMD is a highly prevalent communicable disease primarily caused by EV infections, notably EV-A71 and CV-A16, along with other human EV serotypes (Zhu et al., 2023). Epidemiological surveillance data consistently identify children under 5 years of age as the most vulnerable demographic group, exhibiting the highest disease susceptibility and clinical attack rates (Saguil et al., 2019). With its sophisticated surveillance network, China—the world’s most populous country—has maintained HFMD as a notifiable disease since 2008, accompanied by continuous pathogen surveillance (Esposito and Principi, 2018). Between May 2008 and June 2014, China reported a total of 10,717,283 HFMD cases with 3,046 fatalities, yielding a case fatality rate of 0.03% (Esposito and Principi, 2018). A comprehensive meta-analysis incorporating 23 epidemiological studies revealed that the average incidence rate of HFMD in China stands at 1.61 cases per 1000 population (Chen et al., 2021). In addition to PV vaccines, multiple EV-A71 inactivated vaccine candidates targeting diverse subtypes have progressed through clinical development, with Singapore’s Inviragen (B2 genotype) and Taiwan’s National Health Research Institutes (NHRI) (B4 genotype) advancing their formulations to clinical trials (Chang et al., 2012; Hwa et al., 2013), while three Chinese-developed vaccines from Sinovac Beijing, Vigoo Beijing, and the Chinese Academy of Medical Science (CAMS) (all C4 genotype) have already obtained market approval in China (Lu, 2014; Zhu et al., 2014; Guan et al., 2020; Liu et al., 2021). The Pichia pastoris-expressed EV-A71 virus-like particles (VLP) vaccine maintains authentic viral conformation without genetic material, exhibiting strong immunogenic potential in preclinical evaluations (Wang et al., 2021). The EV-A71 live-attenuated vaccine, engineered through VP1 codon deoptimization combined with high-fidelity 3D^pol^ substitutions, demonstrated potent immunogenicity in murine models by eliciting both cellular and humoral immune responses that conferred complete protection against lethal EV-A71 challenge in neonatal murine models (Hsieh et al., 2024). According to surveillance data from the European Non-Polio Enterovirus Network (ENPEN), a study conducted between 2021 and 2022 identified 10,481 enterovirus-positive samples (6.8% positivity rate) reported by 58 institutions across 19 European countries (Simoes et al., 2024). Among these, 1,004 cases (9.6%) were confirmed as EV-D68 infections. Clinical data analysis of 969 cases revealed that 78.9% of infections occurred in children aged 0–5 years. Inactivated vaccines candidate for EV-D68 have demonstrated the capacity to elicit potent neutralizing antibodies in preclinical animal studies (Zheng et al., 2020; Senpuku et al., 2024). In summary, vaccination remains the cornerstone of enterovirus infection control, with polio immunization programs demonstrating remarkable success in disease elimination. While effective vaccines exist for poliovirus and EV-A71, the persistent circulation of EVs (including environmental poliovirus detection and emerging strains like EV-D68) underscores the need for continued vaccine development, robust surveillance systems, and sustained immunization efforts—particularly for high-risk pediatric populations. The advancement of novel vaccine platforms (e.g., nOPV2, VLPs, and live-attenuated candidates) offers promising strategies to address safety and coverage gaps in current options.

## 6 Conclusions

EVs have evolved sophisticated mechanisms to evade host innate immune responses, ensuring their successful replication and spread. These strategies include the inhibition of PRRs, disruption of key signaling pathways, and direct targeting of IFN signaling components. The use of viral proteases such as 2A^pro^ and 3C^pro^ to cleave host proteins is a common theme in EVs immune evasion. Additionally, EVs manipulate host factors, such as Sox4 and miRNAs, to suppress antiviral responses. Despite these evasion tactics, the development of vaccines against EVs, such as inactivated and live-attenuated formulations, offers promising strategies for controlling infections. Future research should focus on elucidating novel immune evasion mechanisms and developing targeted antiviral therapies to combat EV-induced diseases.
